# Anomalous Origin of the Left Atrial Branch from the Left Main Trunk

**Published:** 2015-04-03

**Authors:** Arash Gholoobi

**Affiliations:** Atherosclerosis Prevention Research Center, Imam Reza Hospital, Mashhad University of Medical Sciences, Mashhad, Iran.

***Text***

A 78-year-old woman was referred for coronary angiography with the chief complaint of exertional angina and dyspnea of a very long duration during routine daily physical activities. She had a history of poorly controlled hypertension and dyslipidemia. Coronary angiography revealed diffuse three-vessel disease. Interestingly, an unusual branch was noted originating from the mid shaft of the left main trunk with a funnel-shaped root and travelling the course of a left atrial (LA) branch (Figures 1 and 2). 

The left main coronary artery (LMCA) usually bifurcates into two major branches: the left anterior descending (LAD) and left circumflex (LCx) arteries. In some patients, the LMCA trifurcates into the LAD, LCx, and a branch artery named ramus intermedius. This third branch arises between the LAD and LCx arteries. The LCx artery gives rise to one or two left atrial circumflex branches which supply the lateral and posterior aspects of the left atrium. According to our extensive search of the literature, this is the first case to be reported with the LA branch originating from the LMCA.

**Figure 1:**
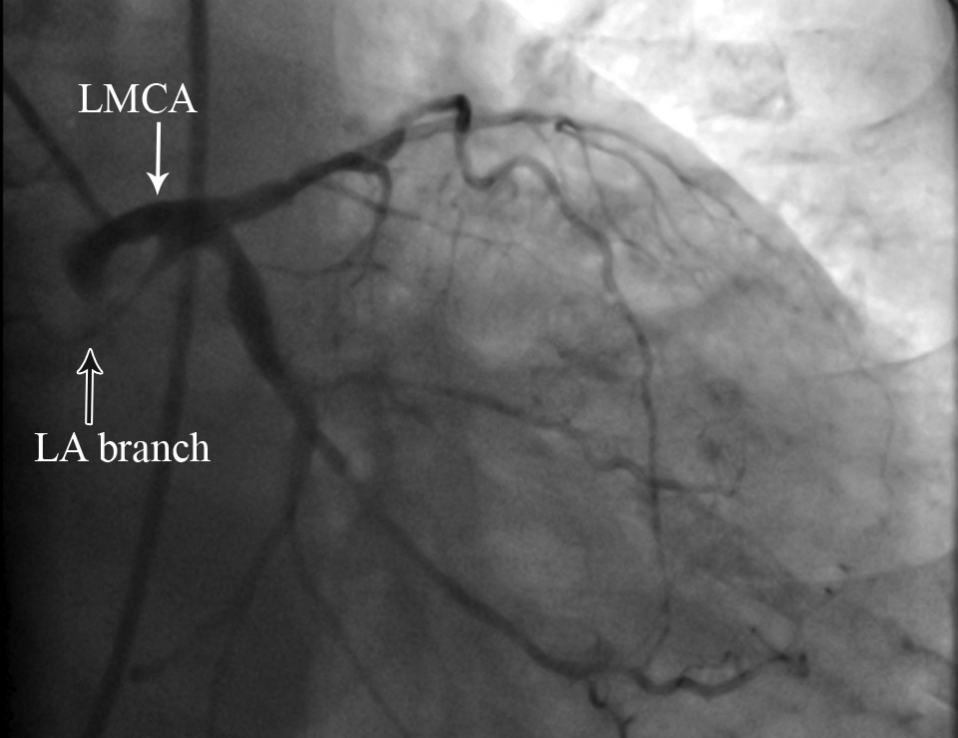
Coronary angiography in the right anterior oblique projection with caudal angulation, demonstrating a branch originating from the left main trunk and traveling the course of a left atrial branch

LA branch, Left atrial branch; LMCA, Left main coronary artery

**Figure 2:**
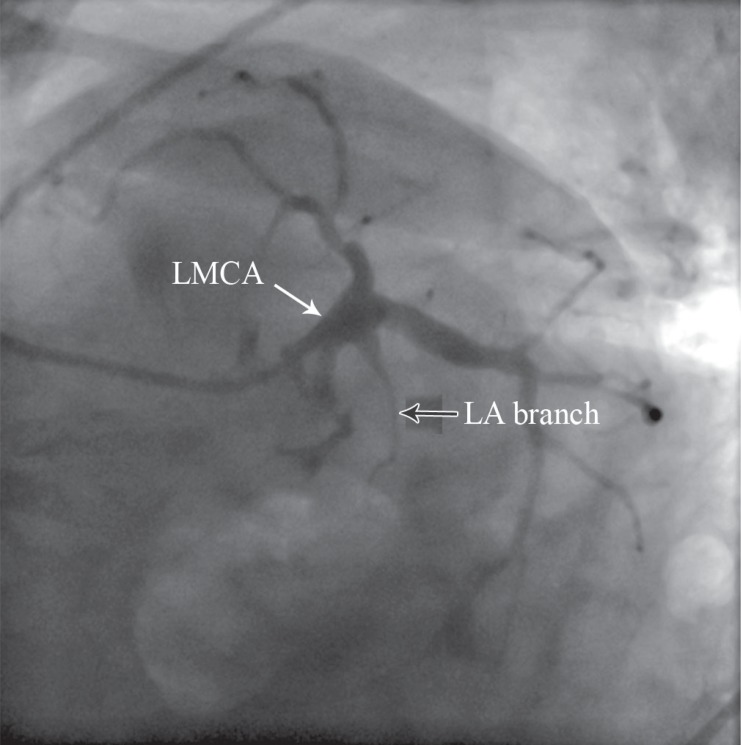
Coronary angiography in the left anterior oblique projection with caudal angulation, demonstrating a branch arising from the left main trunk with a funnel-shaped origin

LA branch, Left atrial branch; LMCA, Left main coronary artery

## Notes:

***This paper should be cited as:*** Gholoobi A. Anomalous Origin of the Left Atrial Branch from the Left Main Trunk. J Teh Univ Heart Ctr 2015;10(2):113-114.

